# A transitional desmosome/tonofibril network may relay mechanical strain to epidermal nerve terminals with high fidelity and sensitivity in the Cuban crocodile (*Crocodylus rhombifer)*: an ultrastructural study

**DOI:** 10.3389/fcell.2026.1739378

**Published:** 2026-02-06

**Authors:** Karl-Gunnar Melkersson, Monika Hodik, Karin Staxäng, Pierre Hakizimana, Hao Li, Helge Rask-Andersen

**Affiliations:** 1 Kolmårdens Tropicarium AB, Kolmården, Sweden; 2 The Rudbeck TEM Laboratory, Uppsala University, Uppsala, Sweden; 3 Department of Biomedical and Clinical Sciences (BKV), Linköping University, Linköping, Sweden; 4 Department of Surgical Sciences, Otorhinolaryngology and Head and Neck Surgery, Uppsala University, Uppsala, Sweden

**Keywords:** crocodilian, desmosomes, ISO receptor, tonofibrils, ultrastructure

## Abstract

**Background:**

Crocodilians are well endowed with multiple cutaneous receptors and specializations, such as integumentary sensory organs (ISOs), which provide formidable mechanical sensitivity despite their protected shield. We investigated the free intraepidermal nerve terminals, focusing on the desmosomes, transitional desmosomes (TDs), corneodesmosomes (CD), and the tonofibril (TF) network that potentially act as force transducers to activate the mechanoreceptors.

**Material and Methods:**

Two Cuban crocodiles (*Crocodylus rhombifer)* were analyzed using light and transmission electron microscopy (TEM) after glutaraldehyde fixation and decalcification.

**Results:**

Discoid nerve terminals were richly enclosed by an epidermal force-transmitting system (e.g., pressure and vibration) through a rigid network of diverse desmosomes and CDs. TDs were anchored to keratinocyte’s cytoskeletons via a dense meshwork of intermediate filaments or TFs, creating a continuous, mechanically-linked web connecting nerve terminals in the epidermis to the stratum corneum. The cutaneous receptors were innervated by myelinated and unmyelinated neural complexes surrounded by thin-walled mesothelial cells.

**Discussion:**

Here, we describe for the first time the ultrastructure of TDs in the crocodile skin with diverse expression of CDs that may focus and amplify force via a tonofibril system “hugging” the receptor. Corneocytes, granular keratinocytes, and nerve endings function as a single integrated system. Thereby, mechanical strain is gathered from a relatively large area of the epidermis and concentrated onto the small surface of the discoid receptor. This may ensure that any deformation of the surrounding corneocytes is efficiently and reliably transferred to the nerve membrane, allowing the crocodile to detect very subtle stimuli. The crocodile system appears to have a far more structured and specialized adaptation for high-fidelity mechanosensation than that of humans.

## Introduction

Crocodiles are endowed with a multitude of highly sensitive skin receptors in their robust armored skin. Specialized cutaneous mechanoreceptors in “integumentary sensory organs” (ISOs) are scattered on the head in Alligatoridae and along the body costume in Crocodylidae and Gavialidae. Physiological measurements have shown their exceptional sensitivity, which may even exceed that of primate fingertips (Leitch and Catania, 2012). Their morphology has been thoroughly studied over the years using both light and transmission electron microscopy (TEM) (von Düring, 1973; Monica von Düring and Miller, 1979; Jackson Butler, 1996; Di-Poï and Milinkovitch, 2013). Free nerve terminals or discoid receptors (DRs) are closely associated with a tonofibril network in the upper layer of the epidermis derived from keratinocyte and transitional desmosomes (TDs). More recent studies have shown that junctional complexes, such as desmosomes, are dynamic and not static (Han Yoon et al., 2001; Windoffer et al., 2011) and may even be integrated parts of a cell signaling pathway where cadherin-based mechanotransduction and keratin intermediate filament dynamics play essential roles (Green and Jones, 1996; Han Yoon et al., 2001; Leckband et al., 2011; Todorović et al., 2013; Hatzfeld et al., 2017; Green et al., 2019). This motivated us to further analyze the fine structure to enhance our understanding of how mechanical force is relayed across skin corneocytes and keratinocytes to the sensory receptors in the outermost layer of the epidermis in the Cuban crocodile (*Crocodylus rhombifer*).

## Materials and methods

Two male juvenile specimens of the Cuban crocodile (*Crocodylus rhombifer*) approximately 1 year of age were anesthetized using Ketamin 5 mg och medetomidin 0.05 mg and euthanized using an intracardial injection of T-61 0.4 mL. Macroscopic images were taken of cranial and abdominal regions using a Samsung Galaxy A56 5G Camera to document ISO receptors. The skin over the maxiallae, mandibulae and abdominal skin were removed and immersed in 2.5% glutaraldehyde and 1% paraformaldehyde (PFA) in 2.5% phosphate buffer. The tissue was placed in 1% osmium tetroxide, dehydrated in graded ethanol, and embedded in Epon. The embedded specimens were divided into different pieces and mounted for semi-sectioning. Sections were stained in toluidine blue and photographed using an Olympus BX63 microscope. Areas of interest were thin-sectioned, and the sections were stained in lead citrate and uranyl acetate and examined at 80 kV in a Tecnai™ G2 Spirit transmission electron microscope (Thermo Fisher/FEI Company, Eindhoven, NL). Images were acquired using an ORIUS™ SC200 CCD camera (Gatan Inc., Pleasanton, CA, United states) using Gatan Digital Micrograph software.

## Results

### LM and TEM

**FIGURE 1:**
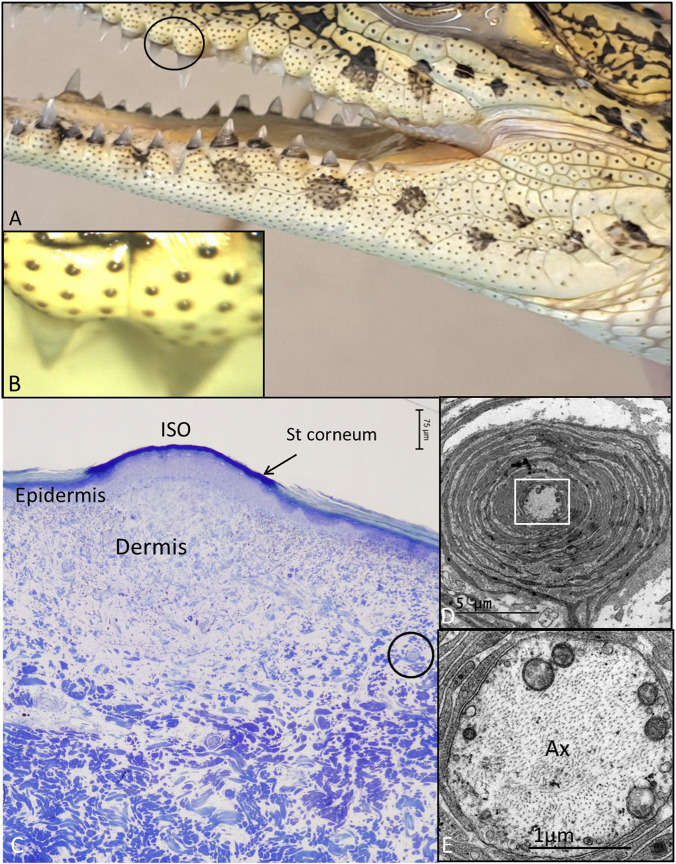
**(A)** Skin ISO receptors in the young Cuban crocodile. Spotted areas with hyperpigmentation (melanocytic areas) can also be seen. **(B)** ISO receptor in the mandibulory skin. **(C)** Light microscopy of a maxillary ISO receptor. A superior layer of specialized keratin stains intensely above the epithelial lining. **(D)** TEM of a lamellar “Pacini” corpuscle located in the dermis [encircled in C]. **(E)** Framed area in D is shown in higher magnification. Ax, axon.

Distribution of pigmented ISO receptors in the head and jaw region of a juvenile Cuban crocodile is shown in Figures 1A,B. A light microscope section shows the dome with a thin, densely stained keratin layer (Figure 1C). The sub-epithelial dermis is loose with denser tissue underneath containing a few encapsulated lamellar or Pacinian corpuscles (Figure 1C). At TEM these had a characteristic appearance with a central axon surrounded by concentric layers of lamellae and connective tissue (Figures 1D,E). At the dome the sub-epithelial tissue also contained melanocytes and an accumulation of iridocytes containing guanine crystals (Supplementary Figure 1).

**FIGURE 2:**
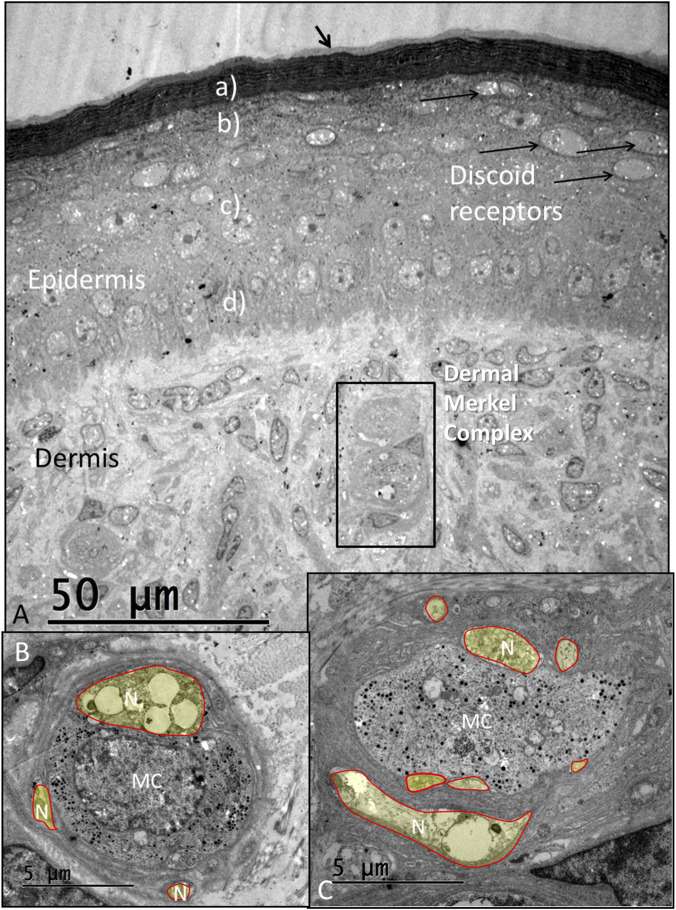
**(A)** TEM showing epidermis and ISO receptor region with electron-dense stratum corneum (a), stratum granulosum (b), stratum spinosum (c) and stratum basale (d). The sub-epithelial layer contains dermal Merkel cell (MC) complexes. Oval discoid receptors are located near the stratum corneum. There is a less densely stained region at the skin surface (black arrow). N, neurons. **(B,C)** Show MCs at higher magnification surrounded by several unmyelinated nerve endings stained in yellow (mandibular skin).

The epidermis consists of an outermost layer of stratum corneum composed of rigid beta-keratin, stratum granulosum, stratum spinosum, and stratum basale (Figure 2A). A thin mucous coat was observed along the outer border of the skin. The granular keratinocyte layer contained many oval-shaped dilated nerve terminals arranged horizontally, similar to those described in the *Caiman crocodilus* skin and often identified as discoid receptors (DR) (von Düring, 1974; Landmann and Villiger, 1975; Monica von Düring and Miller, 1979). They are also present among turtles and lizards (Landmann and Villiger, 1975). These nerve terminals ascend from intra-epidermal unmyelinated nerve fibers passing from the dermis through the epithelium. DRs were also located in neighboring regions but were fewer in number. Encapsulated lamellar corpuscles and Merkel mechanoreceptor cells containing dense-core vesicles were observed in the dermis (Figure 2A, Supplementary Figure 2). These were closely associated with several unmyelinated nerve afferent terminals (Figures 2B,C, Supplementary Figure 3).

**FIGURE 3:**
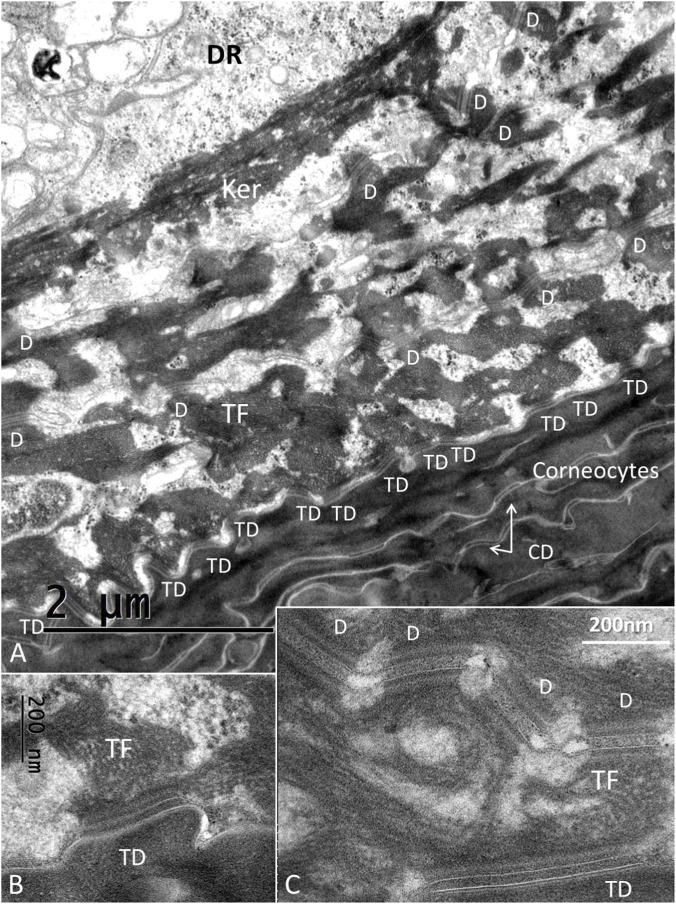
**(A)** TEM of the junction between the corneocyte and granular keratinocyte layers. The interphase is bordered by corneodesmosomes (CDs). Each tonofibril (TF) forms a complex lattice of keratin bundles connecting different keratinocytes (Ker) through desmosomes (D). These bundles form a mantle around the DR perimeter. **(B)** Higher magnification of a TD between a corneocyte and a keratinocyte. TFs extend from the desmosome into the cytoplasm. **(C)** TD and serially connected desmosomes between keratinocytes (Hitachi TEM system). Difference in structure between the inter-keratinocyte D and the TD is noticeable; the TD lacking a cytoplasmic plaque on the corneocyte side (abdominal skin).

**FIGURE 4:**
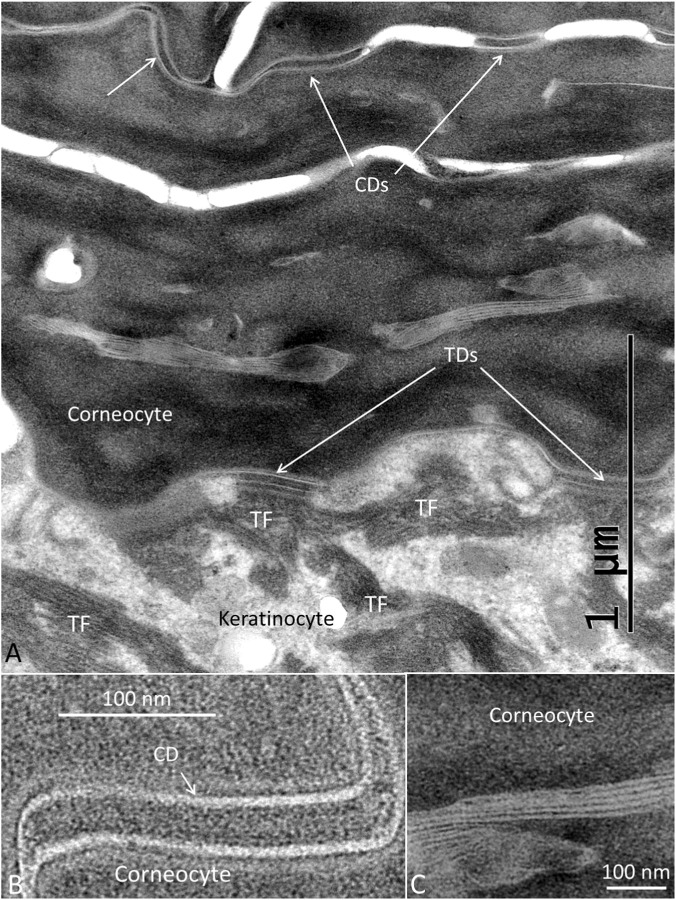
**(A)** TEM of the junction between the stratum corneum and the stratum granulosum. Corneodesmosomes (CDs) are located between corneocyte scales and transitional desmosomes (TD) between keratinocytes and corneocytes. **(B)** Higher magnification of a CD. Scale bar is 100 nm. The central area is around 30 nm. **(C)** Lamellar structures between corneocytes (abdominal skin).

**FIGURE 5:**
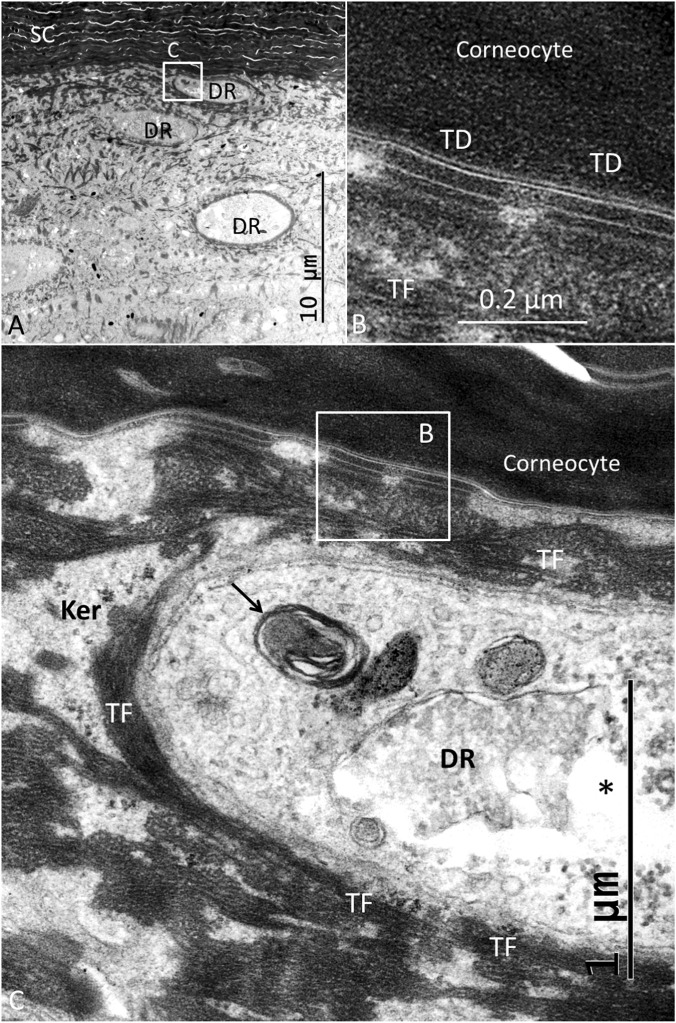
TEM of abdominal skin showing DRs positioned beneath the stratum corneum (SC) **(A)**. Framed area is shown in C **(B)**. Several TDs connect basal corneocytes with the keratinocytes that surround the DR. **(C)**. The DR cytoplasm contains myelin bodies (arrow) and clear and dilated vesicles (*) often filled with glycogen particles. Framed area is magnified in **(B)**. Ker, keratinocyte.

The stratum corneum at the ISO dome consisted of several layers of corneocyte scales forming a protective cutaneous barrier. The corneocytes were composed of anucleate cells containing differently shaped corneodesmosomes (CDs) (Figures 3–5). A layer of transitional desmosomes (TDs) connected and formed adhesive structures between basal corneocytes and the outermost layer of granular keratinocytes (Figures 3B,C). The TDs differed in structure from the typical desmosomes by the lack of a cytoplasmic plaque on the corneocyte side. TDs at the junction between the stratum corneum and the stratum granulosum are also shown in Figure 4A. It also demonstrates the structure of CDs located between corneocyte scales (Figure 4B). Lamellar interconnections between corneocytes are also observed but if these represents modified CDs could not be settled (Figure 4C).

**FIGURE 6:**
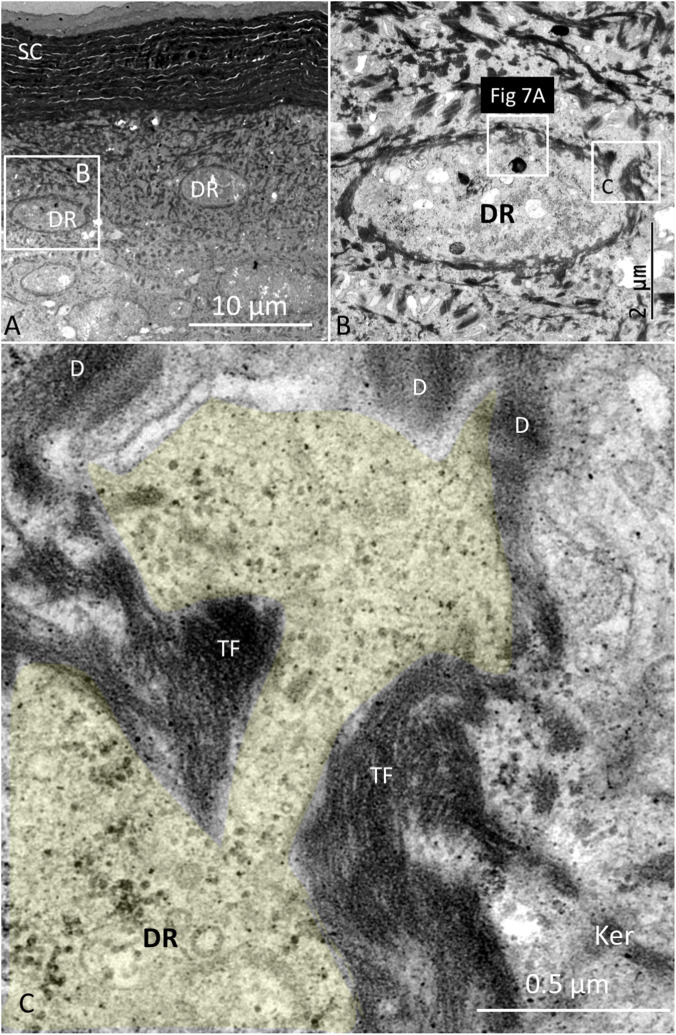
**(A)** TEM of stratum corneum and granular layer containing several DRs in the abdominal skin. SC; stratum corneum. **(B)** Higher magnification of framed area shown in **(A)**. The DR is surrounded by a dark network of tonofibrils (TFs) derived from neighboring keratinocytes. Framed areas are shown at higher magnification in **(C)** and Figure 7A, respectively. **(C)** A cytoplasmic process of the DR (yellow) is closely surrounded by TFs emanating from surrounding keratinocyte desmosomes (D).

**FIGURE 7:**
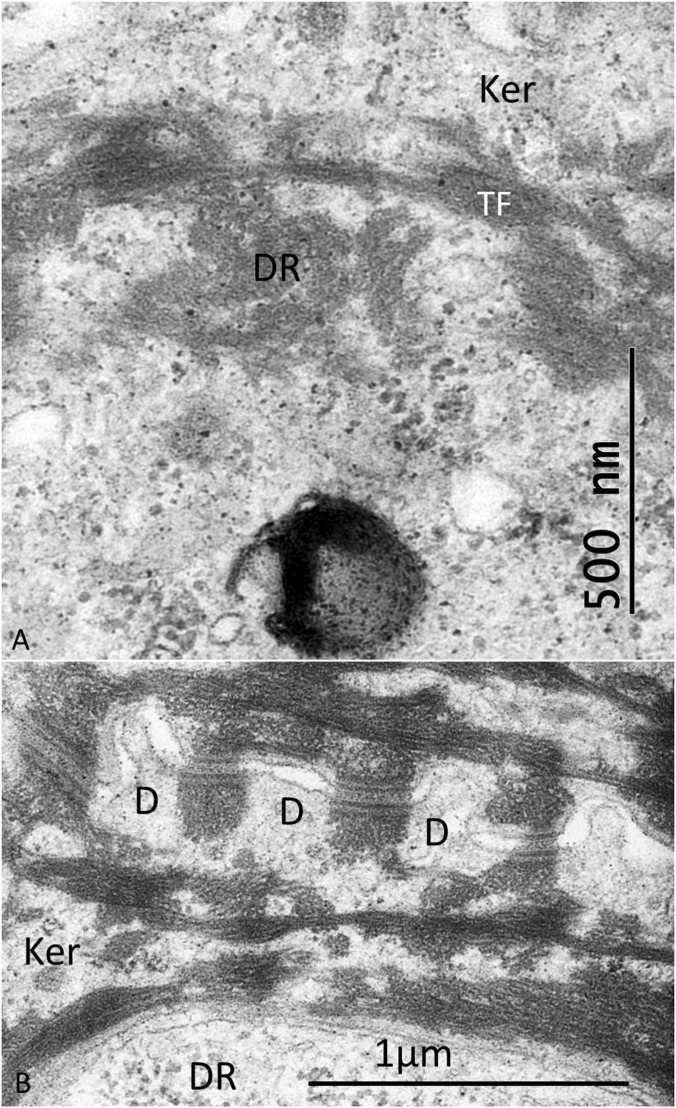
**(A)** Higher magnification of framed area shown in Figure 6B. There is a close association between keratinocyte tonofibrils (TFs) and cytoskeletal elements in the cytoplasm of the discoid receptor (DR). **(B)** Several desmosomes (D) form between adjoining keratinocytes (Ker) with bundles of desmosome tonofilaments and TFs running perpendicularly around the DR.

Their tonofilaments transitioned into bundles crossing the cell cytoplasm, facing their contralateral cell membrane (Figures 5A–C). Several desmosomes connected neighboring keratinocytes, and large bundles of tonofibrils (TFs) were closely associated with the DR plasma membrane. TFs surrounded and “hugged” the DRs with no desmosome plaques (Figures 5–7). Figure 6 shows TEM of stratum corneum and granular layer with several DRs in the abdominal skin. The DR and its neural process are surrounded by a network of TFs originating from surrounding keratinocyte desmosomes (D). There was only a narrow intercellular space separating the keratinocyte from the DR cell membrane. The DR cytoplasm was often vacuolated and contained large amounts of glycogen granules, a few clear vesicles, dense bodies, mitochondria, and occasionally fibrils near the plasma membrane. There were no tight or gap junctions visible between the keratinocytes and the DR cell membranes. Occasionally, keratinocyte TFs appeared to lean against the receptor cell membrane associated with cytoskeletal components with a minimum separating space between them (Figure 7A). Hence, a large variety of different desmosomes seemed to focus and amplify force via the TF system surrounding the DRs onto the small surface of the DRs via the complex interconnecting TF network associated with the DR outer cell membrane surface (Figure 7B).

**FIGURE 8:**
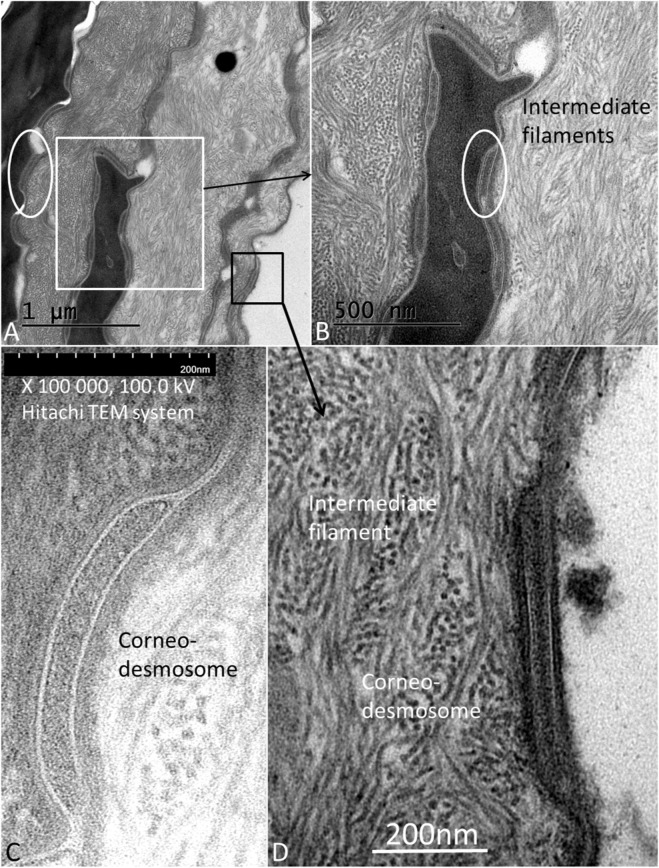
**(A)** TEM of the abdominal skin surface and stratum corneum with layers of intermediate filaments and corneodesmosomes (CDs). White framed area is magnified in **(B)** Black framed area is magnified in **(D)** Encircled areas show CDs positioned between the filamentous layer and corneocyte. **(C)** A CD located at the surface corneocyte (Hitachi TEM system). **(D)** Another desquamated CD overlying the filamentous layer (Tecnai™ G2 Spirit transmission electron microscope).

The surface abdominal skin occasionally displayed a layer of loosely arranged tissue composed of intermediate filaments containing differently shaped CDs of varying sizes assumed of being dispelled (Figure 8).

## Discussion

During their evolution, crocodylians have developed robust keratinized skin for their protection and homeostasis at land dwelling and under aquatic conditions. Nonetheless, molecular and electrophysiological analyses show that their skin is highly sensitive to mechanical, thermal, and pH stimuli but not to osmotic stimuli. This is accomplished through a multisensorial ISO containing neural complexes expressing a set of specific transduction channels (Leitch and Catania, 2012; Di-Poï and Milinkovitch, 2013).

**FIGURE 9:**
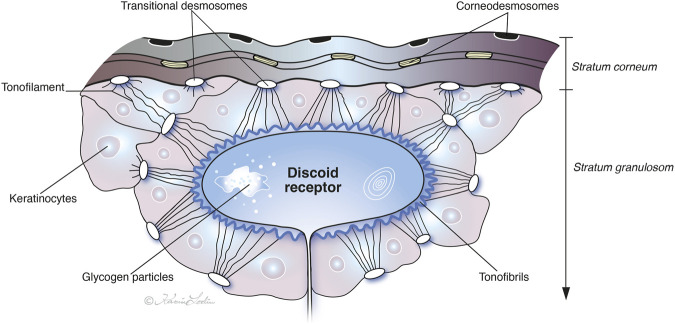
Illustration of the discoid receptor organization and putative nerve triggering in the Cuban crocodile. Free nerve endings are enclosed by keratinocytes with a network of keratin tonofilaments that amalgamate into a tonofibril system (TF) tightly connected to the receptor plasma membrane. Transitional desmosomes (TDs) may transfer mechanical forces from the stratum corneum to the keratinocytes in the stratum granulosum via an array of corneodesmosomes (CDs). (Image by Karin Lodin).

We focused on the free nerve endings located in the granular keratinocyte layer, which are alleged to serve as mechanoreceptors. The present data validate earlier notions that the crocodile possesses a complex epidermal desmosome/tonofibril force-transmitting system in the cutaneous ISO receptors that may stimulate nerve endings as integrated mechanosensing units (Monica von Düring and Miller, 1979). Novel investigations have revealed desmosomes’ roles not only as cell‒cell adhesives to preserve tissue integrity but also as signaling pathways controlling epithelial shape, polarity, function, and modulation of cell behavior (Hatzfeld et al., 2017; Müller et al., 2021). We speculate therefore that the crocodiles desmosomes’ role is to relay external force reception (e.g., pressure and vibration) that deforms the surface of the stratum corneum followed by conduction via a system of CDs and TDs into a keratinocyte TF network surrounding the free nerve endings in the stratum granulosum. This meshwork may act as a mechanical lever system, collecting and focusing force directly onto the intraepidermal nerve ending along desmosome cadherin complexes in response to tension. Different types of desmosomes may anchor the keratinocyte cytoskeleton via the TF system. The keratin intermediate filaments organize into bundles of TFs that exhibit remarkable motile and dynamic properties *in vivo* (Han Yoon et al., 2001). In crocodiles, the desmosome TFs seem to create a continuous, mechanically-linked web that runs through the surrounding keratinocyte. Cadherin proteins within the desmosomes could be critical molecular links in this chain (Kowalczyk and Green, 2013). When desmosomes are displaced, they may pull on the TF network, creating tension. The variety of desmosomes and CDs may focus and amplify gradient force via the TF “hugging” system of the receptor, thereby gathering mechanical strain from a relatively large area of the epidermis and concentrating it onto the small surface of the DR. This may ensure that any deformation of the surrounding keratinocytes is efficiently and reliably transferred to the nerve membrane, allowing the crocodile to detect very subtle stimuli (Figure 9).

The innermost layer of the electron-dense corneocytes was connected to the outermost keratinocytes via a pearl-string-like layer of TDs, from which dense bundles of intermediate filaments coalesce into the keratinocytes´ cytoplasm. TDs may serve as an important bridge to transfer forces from the stratum corneum to the stratum granulosum. TDs lack a cytoplasmic plaque on the corneocyte side and are restricted to the interface between the stratum granulosum and the stratum corneum (Al-Amoudi et al., 2005). Their intercellular midline changes with thickening of the desmosome plaque into electron-dense structures in the stratum corneum. TDs transform into CDs in the stratum corneum, providing strong intercellular adhesion. The extracellular core of CDs has been shown to contain a basic glycoprotein named corneodesmosin, which is specific to cornified epithelia (Matsumoto et al., 2008). CDs seem not only to represent desmosome remnants but also play specific roles in the upper layers of the epidermis (Jonca et al., 2010). The TDs reinforced with proteins such as corneodesmosin are believed to be broken down by proteases to allow for skin shedding or desquamation (Lin et al., 2012). During transition, desmosome plaques fuse, ultimately leading to their degradation and shedding at the surface of the skin.

Mechanical tension generated in the TF system may physically deform the nerve endings’ plasma membrane; a key event that opens mechanosensitive ion channels leading to an influx of ions, depolarization, and ultimately an action potential (Kung et al., 2010; Haswell et al., 2011). Even though large clear vesicles occur in the DR, evidence strongly points to a primarily mechanical activation of the DRs. The direct and extensive physical connection between the TF network and the nerve terminal is the most compelling evidence (Leitch and Catania, 2012). Furthermore, there were no gap junctions, which argues against direct electrical signaling between granular keratinocytes and the nerve. The dense arrangement of TFs “hugging” the receptor is likely crucial for the receptor’s function and sensitivity. There may be two primary functional reasons, such as *force focusing* and *amplification* by the TF network acting to gather mechanical strain from a relatively large area of the epidermis and concentrate it onto the small surface of the discoid receptor. This would significantly amplify the signal, allowing the crocodile to detect subtle stimuli. The tight coupling ensures that any deformation of the surrounding keratinocytes is efficiently and reliably transferred to the nerve membrane, suggesting *a high degree of sensitivity and fidelity* where the keratinocytes and the nerve ending function as a single integrated system.

The presence of larger vesicles in the nerve terminal does not necessarily imply classical chemical neurotransmission. These findings contrast to the Merkel cell complexes in the dermis, which have clear synapse-like structures and dense-core vesicles. This suggests that the crocodile has at least two distinct mechanosensory systems: one based on direct mechanical linkage (DR, Pacini corpuscles) and another (MCs) involving chemical synapses likely for detecting different qualities of touch. Notably, the MCs were located in the dermis layer, unlike in mammals, where they are mostly located at the stratum basale.

Intriguingly, a thin mucous coat was observed along the outer border of the skin, which has also been documented in amphibian skin by Farquhar and Palade (1965). Its role in transmitting mechanical force under different aquatic and terrestrial conditions remains speculative.

### Somatosensory stimulation in crocodile and man–a comparison

Human keratinocytes function as both sensors and transmitters of somatosensory and cutaneous nociception to epidermal receptors (Xu et al., 2022), closely interacting with ensheathing intra-epidermal nerve fibers (Baumbauer et al., 2015; Logan et al., 2024). Keratinocyte encapsulation of afferents and adjacent connexin43 contacts occurs in native skin (Erbacher et al., 2024) but with few ultrastructural descriptions. Tactile stimuli activate the mechanically-gated cationic channel Piezo1, releasing ATP from these cells and activating purinergic P2X4 receptors in sensory afferents (Coste et al., 2010; Mikesell et al., 2022). Gap junctions expressing connexin26 and 43 (Cx26, 43) may play a major role in keratinocyte signaling and afferent nerve communication. Air-stimulated ATP release was increased in calcium differentiated cultures, which showed a corresponding increase in connexin43 mRNA, a major component of keratinocyte hemi-channels (Barr et al., 2013). Signaling also occurs via specialized synapse-like connections (Talagas, 2023), pannexins, or vesicular transport of ATP toward afferent nerve fibers (Sondersorg et al., 2014).

In humans, free nerve endings are a diverse group most commonly associated with nociception (pain) and thermoception (temperature), mediated by ion channels from the transient receptor potential (TRP) family (e.g., TRPV1 for heat/capsaicin). However, some free nerve endings also function as sensitive, low-threshold mechanoreceptors that detect light touch, where mechanical deformation opens mechanosensitive ion channels (like Piezo2) in the nerve membrane. The key difference is that human endings typically lack the highly organized, specialized superstructure of keratinocytes and TFs documented in crocodile ISOs. The crocodile system represents a far more structured and specialized adaptation for high-fidelity mechanosensation. CDs are fundamental components of human stratum corneum—modified desmosomes provide critical cell-to-cell adhesion that creates the protective skin barrier (Murphrey et al., 2022). Their structure and primary function appear conserved in humans and crocodiles.

## Conclusion

Crocodiles seem to have reached the most sophisticated and elegant solutions for achieving high mechanical sensitivity in a tough armored skin. Fine structure organization suggests that a plethora of transitional keratinocyte desmosomes and CD variants gather mechanical strain from a relatively large area of the epidermis onto the small surface of the DR via TFs. Desmosomes are capable of mechanotransduction and play a significant role in sensing and responding to mechanical forces, particularly in epithelial tissues. The desmosome-intermediate filament system may function as a mechanosensing unit, where mechanical stress is focused and amplified via the TF system “hugging” the receptor. Any deformation of the surrounding keratinocytes may be efficiently transferred to the nerve membrane, allowing the crocodile to detect very subtle stimuli. This arrangement seems to work in concert with the more deeply located dermal encapsulated and free nerve endings. The crocodile system appears to be a far more structured and specialized adaptation for high-fidelity mechanosensation than that of humans. The key difference is that in humans, these endings typically lack the highly organized, specialized superstructure of keratinocytes and TFs documented in crocodile ISOs. The advance of these refined and highly specialized sensory skin receptors have allowed crocodilians to discriminate and detect minute motions even under conditions where vision and sound localization are restricted. These unique multi-sensory arrangements have contributed to the crocodilians evolutionary success over millions of years.

## Data Availability

The original contributions presented in the study are included in the article/Supplementary Material, further inquiries can be directed to the corresponding author.

## Supplementary material

SUPPLEMENTARY FIGURE 1
**(A)** TEM of stratum basale (SB) of the epidermis and dermis near an ISO region (maxillary skin). Several pigment cells or melanocytes (Me) and iridocytes (Ir) filled with organic biogenic guanine crystals are seen. **(B)** Iridocyte with many guanine crystals and intracytoplasmic organelles. **(C)** Iridocytes with guanine crystals. **(D)** Higher magnification of a guanine crystal located in an iridocyte.

SUPPLEMENTARY FIGURE 2
**(A)** TEM of a Merkel cell in the lamina propria containing dense-core vesicles (DCVs) associated with an afferent nerve. The mechanoreceptor is surrounded by the basal lamina (BL). **(B)** Synapse-like intercellular complex between the receptor and nerve. **(C)** Higher magnification of the DCVs. **(D)** Zone between the receptor and nerve shows DCVs closely associated with a system of fibrils near the afferent nerve terminal that is rich in glycogen (gly) (maxillary skin).

SUPPLEMENTARY FIGURE 3
**(A)** Dermal nerve complexes supplying receptor corpuscles in the crocodile. Both myelinated nerve fibers (MNFs) and unmyelinated nerve fibers (uMNFs) are surrounded by thin mesothelial cells and collagen (Co) fibers. **(B,C)**. Neural units (arrows) in the dermis containing myelinated and unmyelinated axons. **(D)** RN, Ranvier node. (maxillary skin).
