# Supplementary figures and images for: A transitional desmosome/tonofibril network may relay mechanical strain to epidermal nerve terminals with high fidelity and sensitivity in the Cuban crocodile (Crocodylus rhombifer): an ultrastructural study
Source: Front Cell Dev Biol. 2026 Feb 6;14:1739378. doi: 10.3389/fcell.2026.1739378 (PMC12921410; doi:10.3389/fcell.2026.1739378)

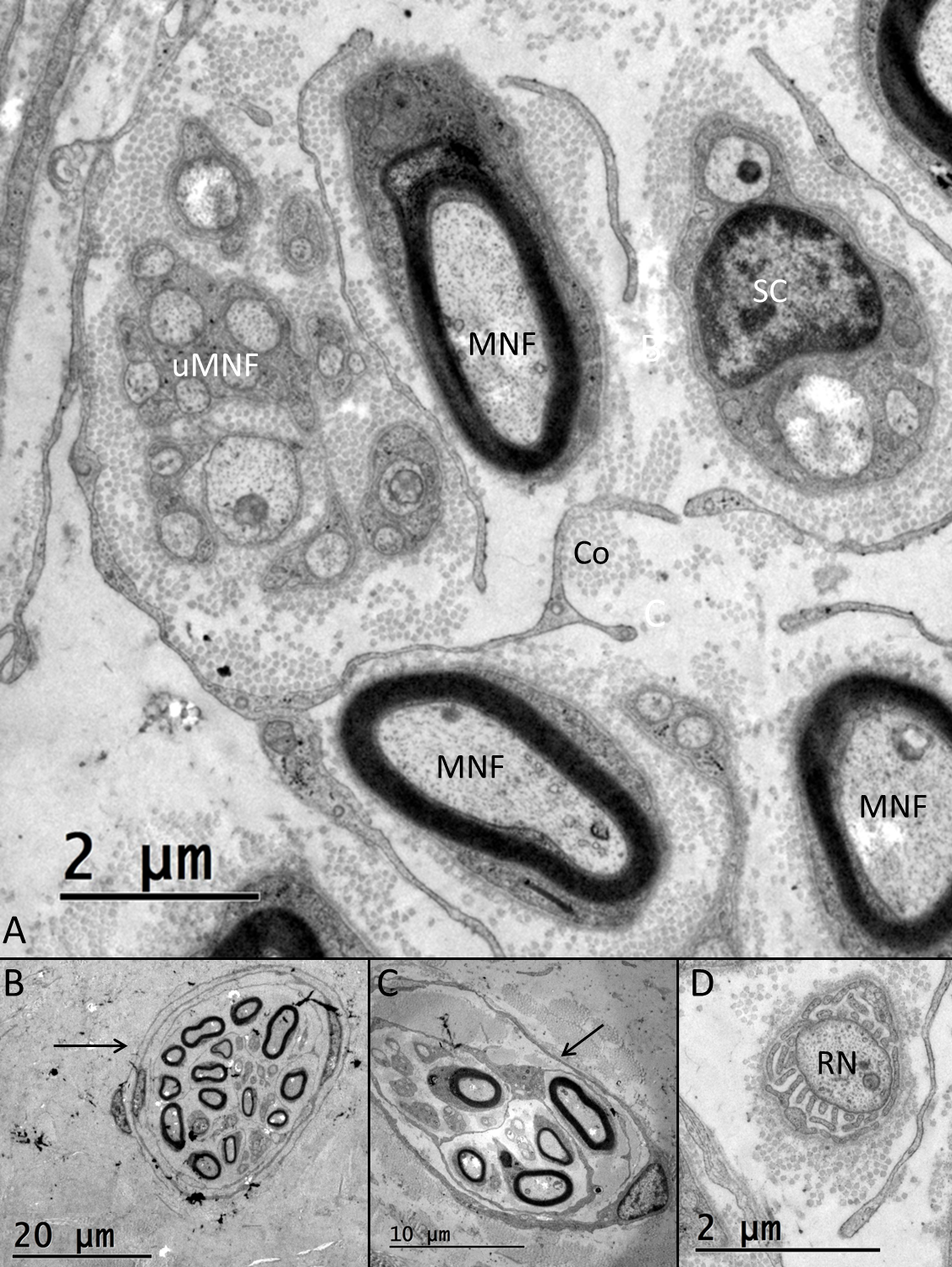

Supplement: Supplementary file 1 [file Image3.tiff]

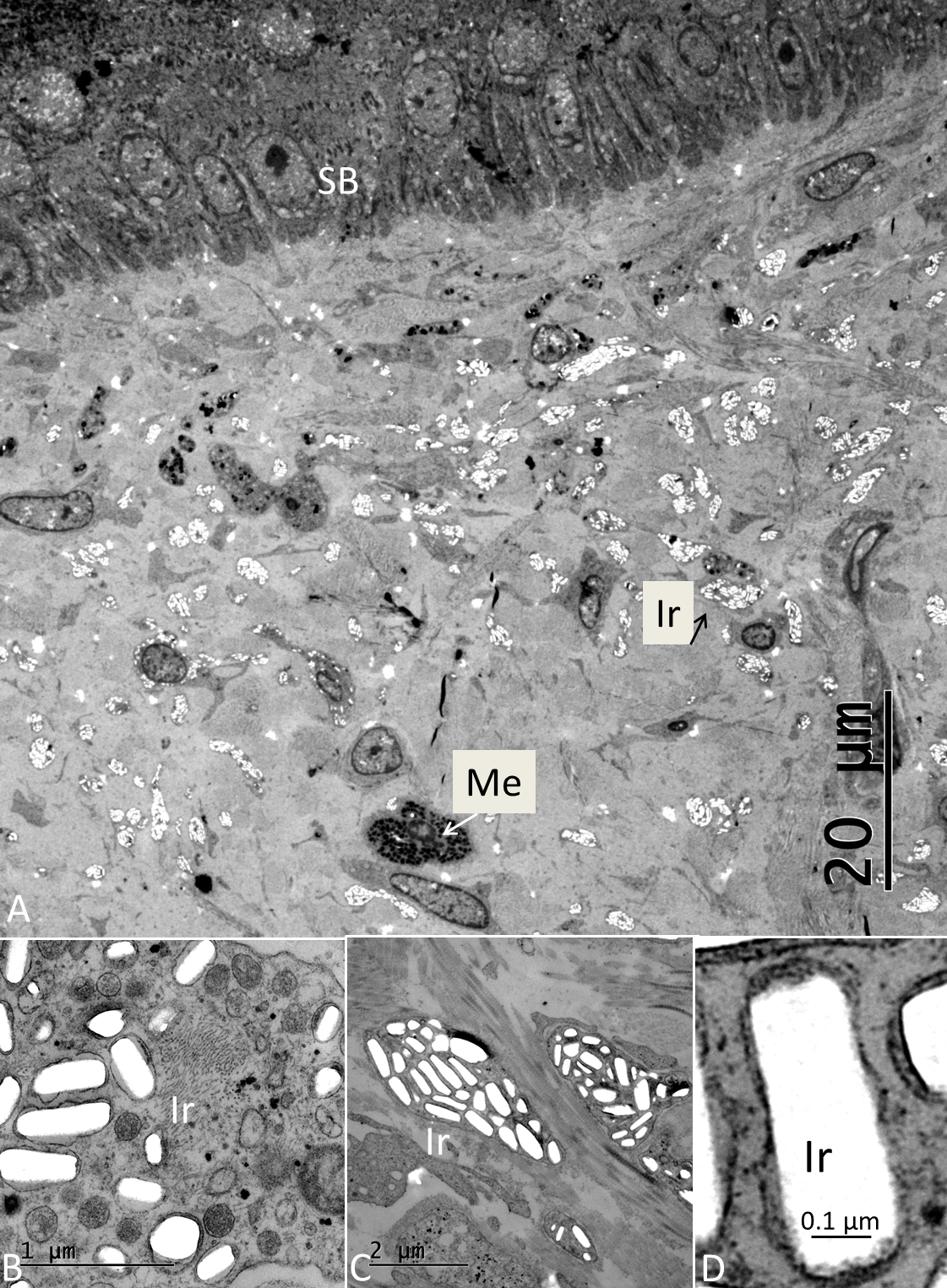

Supplement: Supplementary file 2 [file Image1.tiff]

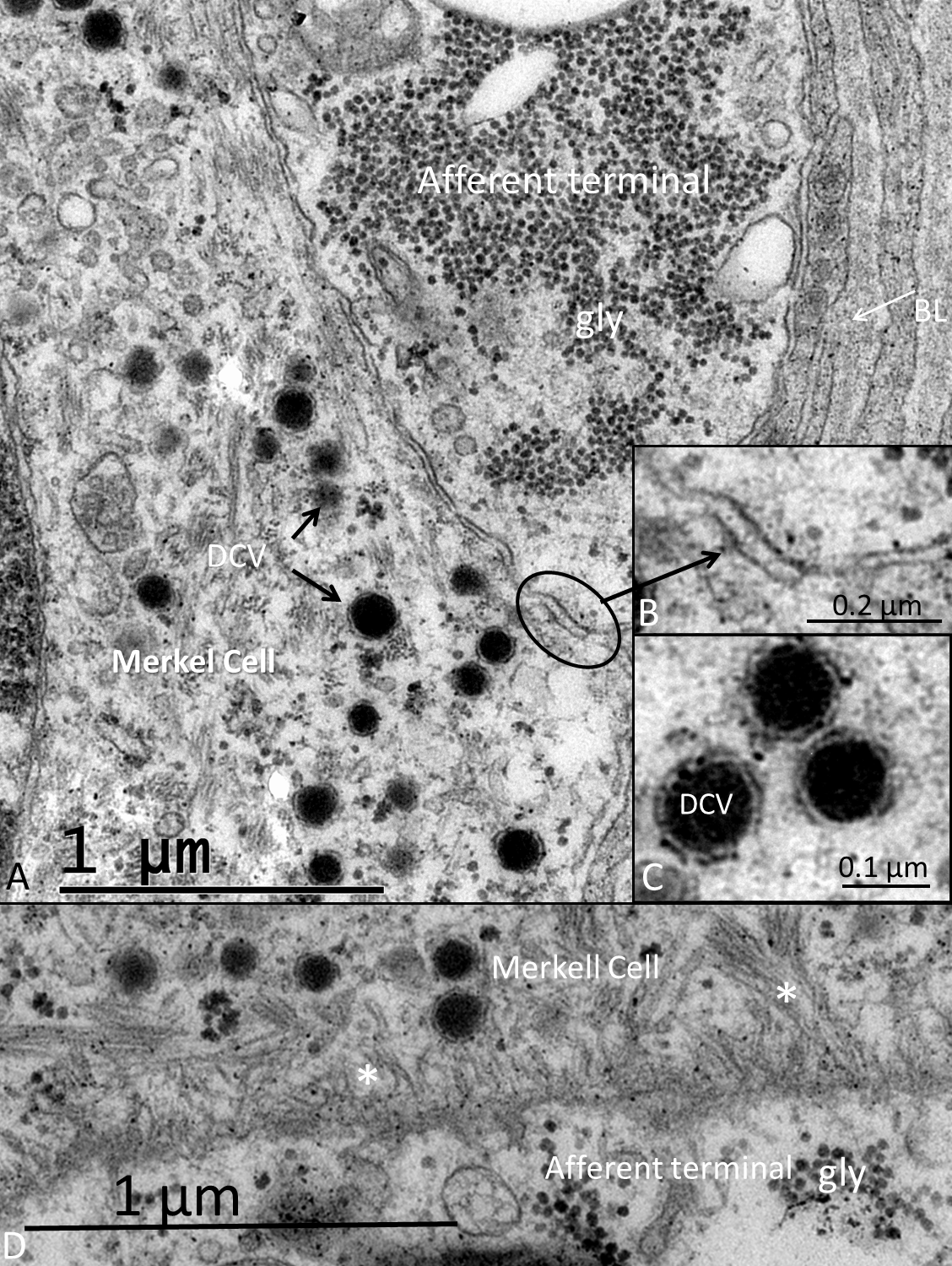

Supplement: Supplementary file 3 [file Image2.tiff]
